# Anti-Atopic Dermatitis Effect of Azalomycin F on 2,4-Dinitrofluorobenzene-Induced Mice and Potential Mechanism

**DOI:** 10.3390/ijms252312846

**Published:** 2024-11-29

**Authors:** Wenjia Zhao, Jianping Zhu, Xinrong Luo, Fengxian Lian, Yanli Yang, Su He, Jinzhou Zhu, Ganjun Yuan

**Affiliations:** 1 Biotechnological Engineering Center for Pharmaceutical Research and Development, Jiangxi Agricultural University, Nanchang 330045, China; 2 College of Animal Science and Technology, Jiangxi Agricultural University, Nanchang 330045, China; 3 Laboratory of Natural Medicine and Microbiological Drug, College of Bioscience and Bioengineering, Jiangxi Agricultural University, Nanchang 330045, China

**Keywords:** atopic dermatitis, Azalomycin F, inflammatory factors, RNA-Seq, NF-κB, TNF

## Abstract

Azalomycin F (AZF) is a kind of antibiotic with antifungal and antibacterial activities, as well as anti-inflammatory and anti-tumor activities. In this study, we evaluated the effects of AZF on atopic dermatitis (AD) and its possible molecular mechanisms. Mice with 2,4-dinitrofluorobenzene-induced AD-like skin lesions were topically treated with 10–30 mg/kg AZF on their dorsal skin for 12 days. Observations focused on skin lesion scores, the frequency of scratching, and histopathological alterations in the skin. In addition, IgE and inflammatory cytokine levels in serum were assessed. The results indicated that topical application of 10–20 mg/kg AZF could reduce skin lesion scores and scratching frequencies in AD mice, while 15–20 mg/kg AZF decreased epidermal thickness and mast cell infiltration. Additionally, the serum levels of IgE, IFN-γ, IL-4, TSLP and IL-1β were reduced with 10–20 mg/kg AZF treatment. Moreover, RNA-Seq was employed to reveal the potential molecular mechanisms underlying anti-AD effects of AZF. KEGG enrichment analysis revealed that the most significantly differentially expressed genes are predominantly enriched in signaling pathways such as NF-κB and TNF. Protein–protein interaction network analysis identifies the key genes including Il1b, Tnf, and Cxcl1. In summary, 15 mg/kg AZF effectively alleviates the inflammatory response in AD mice, and the potential mechanism may involve the regulation of key signaling pathways like NF-κB and TNF, thereby reducing inflammatory factor levels and eliciting an anti-inflammatory effect. These findings provide valuable scientific evidence for the development of novel natural drugs for the treatment of AD.

## 1. Introduction

Atopic dermatitis (AD), a chronic inflammatory skin condition marked by eczematous lesions and persistent itching, is often linked to other atopic conditions such as allergic rhinitis and asthma [1]. Over recent years, the prevalence of AD has surged globally, affecting an estimated 200 million individuals. The adult incidence rate ranges from 1 to 3%, while in children, it is significantly higher, at 10–20%. In China, the rate among children is notably around 12.94% [2,3,4]. It is noteworthy that the COVID-19 pandemic has further exacerbated the situation for healthcare workers, with nearly 97% experiencing some form of skin damage due to occupational hazards and preventive measures, some cases of which have escalated to AD [5,6]. As a global public health issue, the etiology and pathogenesis of AD are extremely complex, involving multiple levels, including genetic and epigenetic susceptibility [7], skin microbiome abnormalities [8], skin barrier dysfunction [9], immune imbalance and inflammatory responses, as well as environmental factors [10]. In addition, the abnormal activation of immune cells, cytokines, and signaling pathways plays a pivotal role in the pathogenesis of AD [11,12,13,14]. Moreover, the infection of *Staphylococcus aureus* has also been confirmed to be closely related to the onset of AD, providing new directions for the treatment of this disease [15]. Currently, the clinical treatment of AD primarily relies on medications such as topical corticosteroids and local calcineurin inhibitors to alleviate the patient’s inflammatory response. However, long-term use of these drugs may lead to side effects such as skin atrophy, hypertrichosis, pigmentation, and drug dependency [16]. In recent years, biological agents have demonstrated remarkable efficacy in AD treatment, yet their significant costs have limited their widespread utilization [17]. Therefore, the research and development of novel, safe, effective, and economically viable therapeutic strategies are crucial in the field of AD. Natural medicines and their derivatives have attracted attention due to their distinctive pharmacological properties, lower toxicity, and abundant resources. An increasing number of studies are now investigating the potential application of these substances in the treatment of chronic inflammatory diseases, aiming to provide patients with safer, more effective, and economical treatment options.

Azalomycin F (AZF) is a class of 36-membered polyhydroxy macrolide antibiotics derived from the secondary metabolites of streptomyces, which exhibit significant antibacterial activity, especially against Gram-positive bacteria like *Candida albicans*, *Staphylococcus aureus*, and *Bacillus subtilis* [18]. Research has shown that AZF exhibits antimicrobial, anticancer, and anti-inflammatory bioactivities, positioning it as a promising therapeutic candidate for a wide range of diseases [19,20,21,22,23]. Zhong et al. discovered that AZF_4a_ can effectively inhibit autophagy in tumor cells by targeting the overexpressed autophagy-related gene 4B (ATG4B) in advanced gastric cancer, thereby possibly providing a new agent for the treatment of this disease [21]. Using the λ-carrageenan-induced rat paw edema and pleurisy models, He, et al. discovered that intraperitoneal injection of AZF exhibits anti-inflammatory activity by effectively reducing the circumference of the rat’s paw and decreasing the volume of pleural effusion [22]. Preliminary research conducted by our research group showed that topical application of AZF can significantly improve skin lesion symptoms in mice with atopic dermatitis and lower the levels of serum inflammatory factors IL-4 and IgE [23]. However, its application in the treatment of inflammatory diseases and the underlying molecular mechanisms remain inadequately described. Currently, clinical research on AZF primarily focuses on the treatment of infections caused by *Trichomonas* and *Candida albicans*, and no related drugs have yet been clinically approved [24]. Based on these, we hypothesize that AZF may play a potential role in treating inflammatory diseases and reducing the risk of AD. In this study, a mouse model of AD-like reactions induced by 2,4-dinitrofluorobenzene (DNFB) was utilized to explore the inhibitory effect of AZF on mouse AD-like reactions through repeated topical applications. Furthermore, transcriptomics methods were employed to delve deeper into the potential molecular mechanisms underlying the effect of AZF on AD.

## 2. Results

### 2.1. Effect of AZF Treatment on DNFB-Induced AD-like Clinical Symptoms

**Figure 1:**
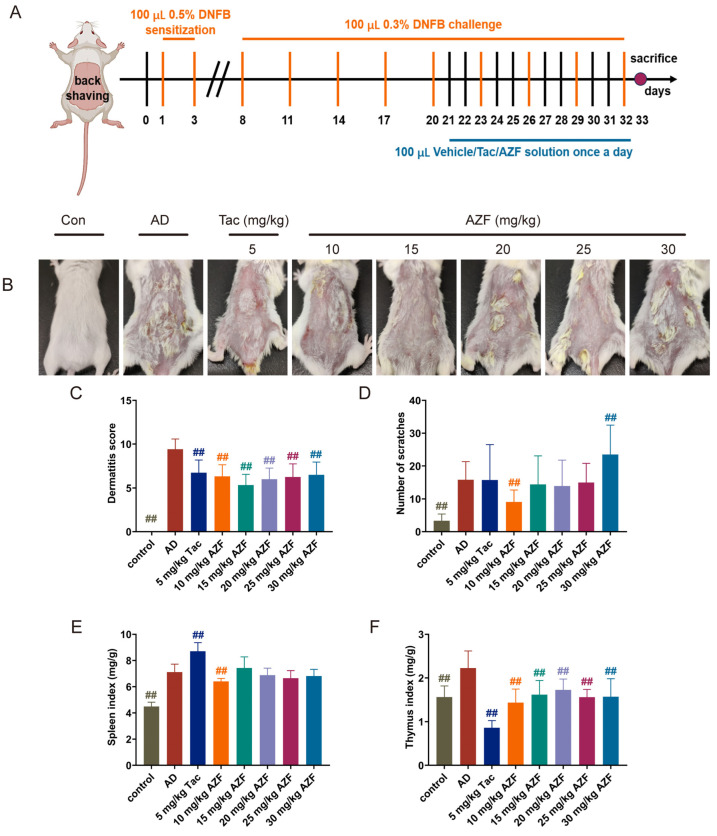
Effects of Azalomycin F (AZF) on atopic dermatitis (AD)-like clinical symptoms in 2,4-dinitrofluorobenzene (DNFB)-induced Balc/c mice. (**A**) Schematic diagram of the experimental protocol. (**B**) Images of the dorsal skin of mice in each group. (**C**) Dermatitis score. (**D**) The number of scratches by mice within 15 min. The results are expressed as the mean ± SD (*n* = 12). (**E**) spleen index and (**F**) thymus index. The results are expressed as the mean ± SD (n = 6). Differences were assessed by analysis of variance (ANOVA) and denoted as follows: ^##^ *p* < 0.01, compared to AD group. Tac, tacrolimus.

In this study, by repeatedly applying a solution of DNFB locally, an AD-like reaction characterized by erythema, excoriation, crusting, epidermal thickening, and dry desquamation was successfully induced on the dorsal skin of mice. After treatment with AZF, the AD-like reaction in mice was improved (Figure 1B). Compared with the control group, the skin lesion score of the AD group mice was significantly increased (*p* < 0.01); compared with the AD group, 10–30 mg/kg AZF and Tac significantly reduced the skin lesion score of the mice (*p* < 0.01, Figure 1C). Furthermore, as an important indicator for assessing the severity of AD, the scratching behavior of the AD group mice increased significantly compared with the control group (*p* < 0.01), and the administration of 10 mg/kg AZF significantly reduced the scratching frequency of the mice (*p* < 0.01, Figure 1D), effectively alleviating their itching sensation. In addition, considering the key role of the abnormal activation of the immune system in the pathogenesis of AD, this study further evaluated the organ indices of the spleen and thymus, two important immune organs. Compared with the control group, the indices of the spleen and thymus in the AD group mice were significantly increased (*p* < 0.01), indicating an overactivation of the immune system. Ten mg/kg AZF significantly reduced the spleen index, and AZF at doses of 10, 25, and 30 mg/kg significantly reduced the thymus index in the AD group mice (*p* < 0.01, Figure 1E,F), suggesting that AZF has a positive effect on regulating the immune function of mice with AD. 

### 2.2. Effect of AZF Treatment on Histopathology of Skin Tissue

**Figure 2:**
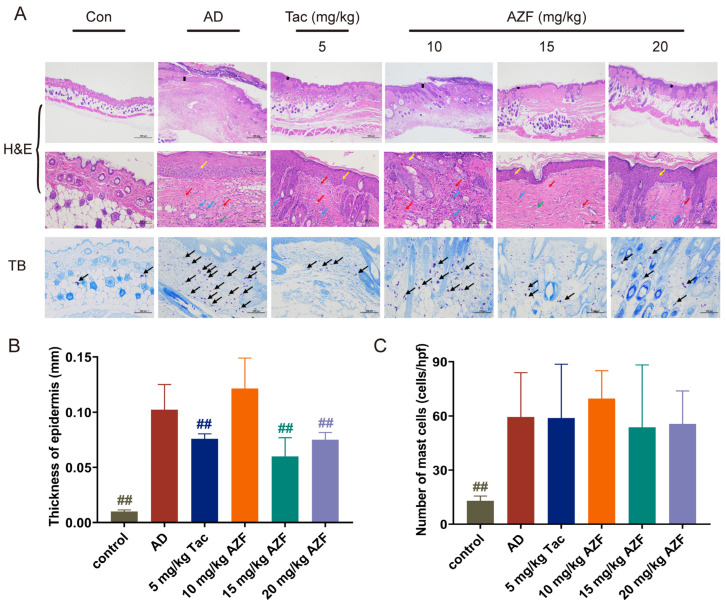
Effects of Azalomycin F (AZF) on DNFB-induced AD-like histopathological alterations in Balb/c mice. (**A**) Representative images of hematoxylin eosin (H&E) and toluidine blue (TB) staining in each group. H&E-stained specimens were observed under a microscope at 40× and 200× magnification (Scale bar = 500 and 100 μm). TB-stained specimens were observed under a microscope at 200× magnification (Scale bar = 100 μm). Black lines indicate the thickness of the epidermis, yellow arrows denote epidermal thickening, red arrows point to neovascularization, blue arrows represent inflammatory cells, green arrows indicate connective tissue hyperplasia, and black arrows denote mast cells. (**B**) The epidermal thickness of each group. (**C**) The number of mast cells in each group. The results are expressed as the mean ± SD (n = 3). Differences were assessed by analysis of variance (ANOVA) and denoted as follows: ^##^ *p* < 0.01, compared to AD group.

Pathological analysis of the dorsal skin of mice in each group was conducted using H&E staining. The results showed that the skin structure of mice in the control group was intact and clear, with a thinner epidermis, neatly arranged connective tissue in the dermis, and scattered distribution of skin appendages such as hair follicles and sebaceous glands, with no obvious necrosis or inflammatory cell infiltration. In contrast, the skin structure of mice in the AD group showed significant abnormalities, with increased epidermal thickness (*p* < 0.01), incomplete or excessive keratinization of the stratum corneum, thickening of the dermis, spongiotic edema, infiltration by a large number of neutrophils and lymphocytes, a decrease in the number of skin appendages such as hair follicles and sebaceous glands, and the appearance of a few new blood vessels (red arrows) (Figure 2A, upper and middle panel; Figure 2B). Compared with the AD group, the skin tissue of mice in the Tac group showed a reduction in epidermal thickness (*p* < 0.01), improvement in incomplete or excessive keratinization of the stratum corneum, a decrease in lymphocyte infiltration, and an increase in the number of skin appendages such as hair follicles and sebaceous glands; mice in the 15 and 20 mg/kg AZF groups showed a reduction in epidermal thickness (*p* < 0.01), improvement in incomplete or excessive keratinization of the stratum corneum, a decrease in lymphocyte infiltration, improvement in skin inflammation, and an increase in the number of skin appendages such as hair follicles and sebaceous glands (Figure 2A, top panel; Figure 2B). Further observation with TB staining revealed that treatment with 15 and 20 mg/kg AZF slightly reduced the infiltration of mast cells in the AD model mice (Figure 2A, lower panel; Figure 2C).

### 2.3. Effect of AZF on Serum IgE and Inflammatory Mediators

The pathogenesis of AD involves the abnormal activation of the immune system, including T cells and inflammatory signaling pathways such as Th1, Th2, Th17, and Th22. The balance of Th1/Th2 immunity in the body can be assessed by monitoring the levels of interferon-γ (IFN-γ) and interleukin-4 (IL-4) in the serum of mice. Additionally, interleukin-1β (IL-1β), as a key factor in innate immunity, plays a central role in the development of the acute phase of AD. At the same time, thymic stromal lymphopoietin (TSLP), as an alarmin, is produced by epithelial cells and promotes the synergy between innate and adaptive immunity, activating the immune response to external threats. 

**Figure 3:**
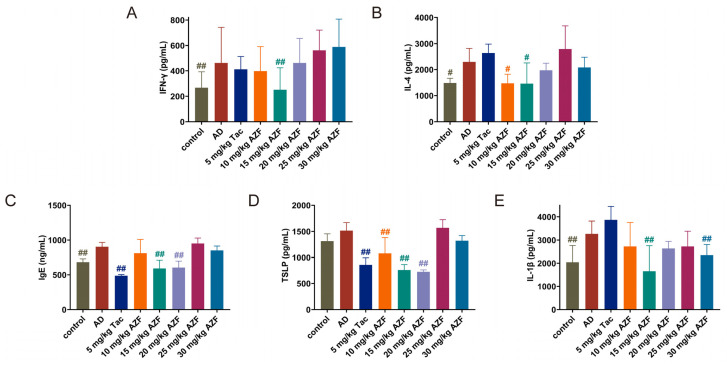
Effects of azalomycin F (AZF) on IgE and inflammatory mediator levels in the serum of DNFB-induced Balb/c mice. (**A**) IgE, (**B**) IFN-γ, (**C**) IL-4, (**D**) TSLP, and (**E**) IL-1β. The results were expressed as the mean ± SD (n = 5~6). Differences were assessed by analysis of variance (ANOVA) and denoted as follows: ^#^ *p* < 0.05, compared to AD group; ^##^ *p* < 0.01, compared to AD group.

Compared with the control group, the levels of IFN-γ in the serum of the AD group mice were significantly elevated, and after treatment with 10 and 15 mg/kg AZF, the expression levels of serum IFN-γ were reduced (Figure 3A). Similarly, the level of IL-4 in the serum of the AD group mice was elevated compared with the control group, and after treatment with 10, 15, 20 and 30 mg/kg AZF, the expression levels of serum IL-4 were reduced (Figure 3B). These results showed that the balance of Th1/Th2 immune response in the body was re-established, mainly by inhibiting the Th2 cytokine IL-4. The literature reports that the reduction in the IL-4 cytokine can further decrease the expression of immunoglobulin E (IgE) levels after B cell activation, and the expression of IgE levels is mainly used clinically to reflect the severity of allergic reactions in the body [25]. Compared with the AD group, 15 and 20 mg/kg AZF significantly reduced the levels of IgE in the serum of mice, alleviating the allergic reactions and inflammatory levels of AD mice (Figure 3C). In addition, compared with the AD group, the levels of alarmin TSLP and pro-inflammatory cytokine IL-1β in the serum of mice in the 15 mg/kg AZF treatment group were significantly reduced (Figure 3D,E). The above findings suggest that one of the possible mechanisms of the anti-AD effect of 15 to 20 mg/kg AZF is to reduce the degree of allergy and inflammatory response in AD mice by restoring Th1/Th2 immune abnormalities and reducing the expression of inflammatory factors in serum (Figure 3).

### 2.4. RAN-Seq Date Analyses

To deeply explore how AZF mitigates the AD-like reactions induced by DNFB in mice and its molecular mechanisms, RNA was extracted from skin tissue samples of mice in Control, AD and 15 mg/kg AZF groups for RNA-Seq analysis, which were successively named Con, AD and AZF group.

Sequencing of each sample yielded more than 36.55 million raw reads, and after filtering, more than 35.71 million clean reads were obtained, with the proportion of base quality exceeding Q20 and Q30 being at least 93.89% (Supplementary Table S1). The principal component analysis (PCA) plot for the three groups showed that PC1 and PC2 accounted for 39% and 23% of the total variation among the nine samples, respectively, indicating a clear separation between the samples (Supplementary Figure S1A). The above sequencing data and results demonstrate that the RNA sequencing data quality control is well managed, with good intra-group repeatability and inter-group discrimination, meeting the requirements for subsequent bioinformatics analysis.

**Figure 4:**
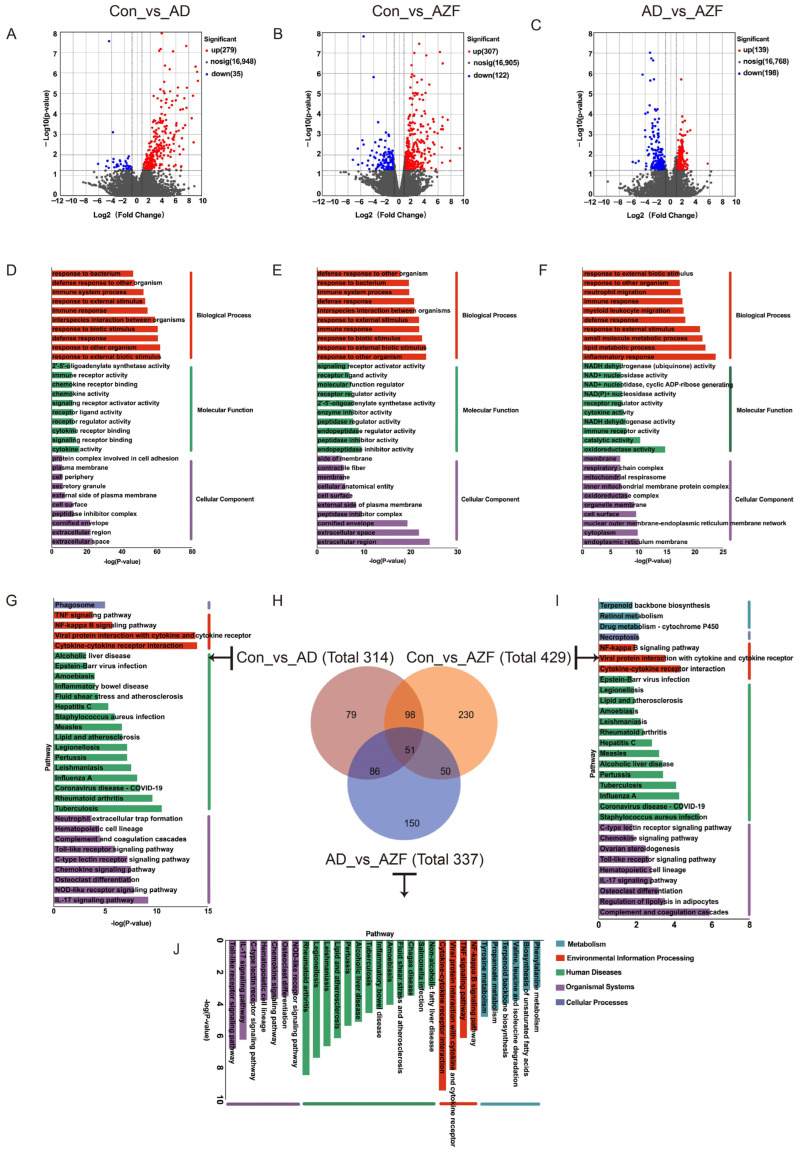
The bioinformatics analyses identified differentially expressed genes (DEGs) and enriched pathways. (**A**–**C**) Volcano plot of DEGs in the Con_vs_AD, Con_vs_AZF and AD_vs_AZF group. (**D**–**F**) Gene Ontology (GO) annotation of DEGs in the Con_vs_AD, Con_vs_AZF and AD_vs_AZF group. The top 10 functional classified GO terms of DEGs annotated by the subontology of GO analysis including Biological Process (BP), Molecular Function (MF), and Cellular Components (CC). (**G**,**I**,**J**) The top 30 pathways with the most significant Kyoto Encyclopedia of Genes and Genomes (KEGG) enrichment in the Con_vs_AD, Con_vs_AZF and AD_vs_AZF group. (**H**) Venn diagrams of DEGs. CON: control group; AD: atopic dermatitis model group; AZF: 15 mg/kg AZF group.

EdgeR was employed to screen for differentially expressed genes. Compared with the Con group, the AD and AZF groups were identified to have 314 differential genes (with 279 genes upregulated and 35 genes downregulated) and 429 differential genes (with 307 genes upregulated and 122 genes downregulated), respectively; when comparing the AZF group to the AD group, a total of 377 differential genes were identified (with 139 genes upregulated and 198 genes downregulated) (Figure 4A–C). Subsequently, a cluster analysis of the differential genes revealed that samples from the same treatment group showed higher similarity, and the three biological replicates of each sample were clustered together with almost identical distances, indicating good repeatability within each sample (Supplementary Figure S1B). The co-expression analysis of differential genes between Con_vs_AD, Con_vs_AZF, and AD_vs_AZF identified 137 co-expressed differential genes common to both Con_vs_AD and AD_vs_AZF (Figure 4H). Interestingly, among these, 129 differential genes were upregulated in the Con_vs_AD comparison group and downregulated in the AD_vs_AZF comparison group; 5 differential genes were downregulated in the Con_vs_AD comparison group and upregulated in the AD_vs_AZF comparison group (Table 1).

### 2.5. Go Annotation and KEGG Enrichment

To further understand the functions of the DEGs and determine their roles at the molecular level, functional analysis was conducted on the differential genes, and they were enriched in the GO and KEGG databases. The GO functional analysis mainly includes three parts: Cellular Component (CC), Molecular Function (MF), and Biological Process (BP). The top 10 most significantly enriched GO terms in the Con_vs_AD, Con_vs_AZF, and AD_vs_AZF groups were statistically analyzed. In the Cellular Component (CC), the differential genes in the Con_vs_AD and Con_vs_AZF groups were mainly annotated to the extracellular space and extracellular region, while the differential genes in the AD_vs_AZF group were annotated to the endoplasmic reticulum membrane. At the Molecular Function level (MF), the differential genes in the Con_vs_AD, Con_vs_AZF, and AD_vs_AZF groups were mainly annotated to cytokine activity, endopeptidase inhibitor activity, and oxidoreductase activity, respectively. At the Biological Process level (BP), the DEGs in each group were mainly annotated to responses to stimuli and immune inflammatory responses, with the differential genes in the Con_vs_AD and Con_vs_AZF groups mainly annotated to responses to other organisms, and the differential genes in the AD_vs_AZF group annotated to inflammatory responses (Figure 4D–F).

Subsequent analysis further performed KEGG pathway enrichment analysis on the differentially expressed genes to identify pathways that significantly changed under different treatment conditions. All DEGs were enriched according to cellular processes, environmental information processes, human diseases, metabolism, and organismal systems. The DEGs in the Con_vs_AD group were mainly involved in the following three classification pathways: (1) Signaling pathways related to the immune system, including NOD-like receptor signaling pathway, IL-17 signaling pathway, Chemokine signaling pathway, C-type lectin receptor signaling pathway, Toll-like receptor signaling pathway, Complement and coagulation cascades, Hematopoietic cell lineage, Neutrophil extracellular trap formation, Cytosolic DNA-sensing pathway, etc.; (2) Signaling pathways related to infectious diseases, including Pertussis, Legionellosis, Yersinia infection, Staphylococcus aureus infection, Tuberculosis, Coronavirus disease—COVID-19, Leishmaniasis, etc.; (3) Signaling pathways related to signal transduction, including NF-κB signaling pathway, JAK-STAT signaling pathway, and TNF signaling pathway (See Supplementary Table S2, Figure 4G). Since the Con_vs_AD group and the Con_vs_AZF group share 149 DEGs, the pathways enriched between them are similar, all related to the immune system, infectious diseases, and signal transduction (See Supplementary Table S3, Figure 4I). In addition, by comparing the KEGG enrichment pathways of the Con_vs_AD and AD_vs_AZF groups, 34 pathways were found to intersect (Table 2, Figure 4J). Among them, pathways related to the immune system include NOD-like receptor signaling pathway, IL-17 signaling pathway, Chemokine signaling pathway, C-type lectin receptor signaling pathway, Toll-like receptor signaling pathway, Complement and coagulation cascades, etc., but the B cell receptor signaling pathway, Th17 cell differentiation, and Intestinal immune network for IgA production are unique to the AD_vs_AZF group. Also, two common signal transduction pathways were enriched—TNF signaling pathway and NF-κB signaling pathway—while the MAPK signaling pathway, HIF-1 signaling pathway, and AMPK signaling pathway were unique to the AD_vs_AZF group, and the JAK-STAT signaling pathway was unique to the Con_vs_AD group. Some infectious-disease-related signaling pathways were also enriched, such as Pertussis, Legionellosis, Yersinia infection, Staphylococcus aureus infection, Tuberculosis, Coronavirus disease—COVID-19, Leishmaniasis, etc. (Supplementary Table S4).

### 2.6. PPI Network Construction and Module Analysis

**Figure 5:**
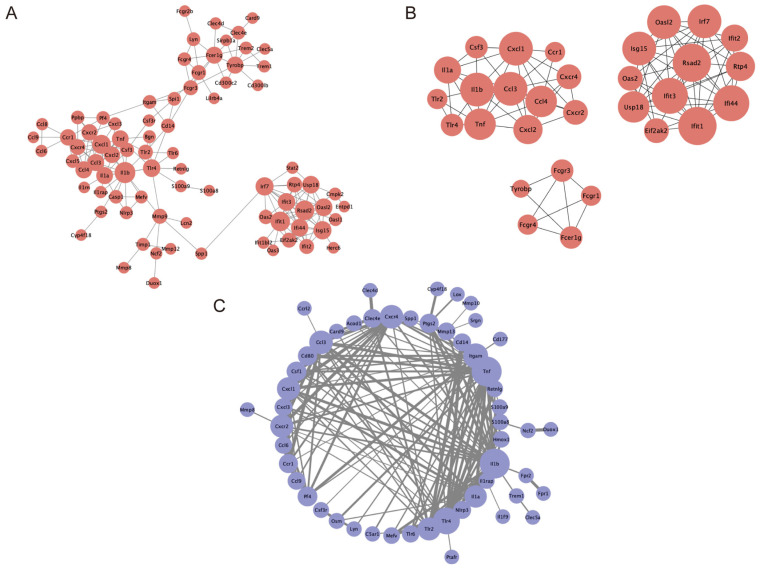
Protein–protein interaction (PPI) network construction of DEGs. (**A**) The PPI network of DEGs identified from Con_vs_AD. (**B**) The top 30 highly connected DEGs and the top 3 significant modules extracted from the PPI network (MCODE scores are 15 and 13). (**C**) PPI network constructed from 137 co-expressed genes between Con_vs_AD and AD_vs._AZF. Red represents gene expression upregulated in (**A**,**B**). Node size indicates the number of genes interacted with in (**A**,**C**). Line thickness indicates the strength of data support in (**C**).

To further investigate the interactions among DEGs, we constructed a protein–protein interaction (PPI) network for the differential genes in the Con_vs_AD group. This network map included genes related to Il1b, Cxcl1, Tnf, Tlr2, Tlr4, Cd14, Cxcl3, Tlr6, Lyn, etc. (Figure 5A). The cytoHubba plugin identified the top 30 highly connected genes (Hub genes), forming three independent networks (Figure 5B), which correspond to the four significant modules identified by the MCODE plugin. These three independent networks consisted of 13 nodes with 40 edges, 12 nodes with 50 edges, and 5 nodes with 8 edges, respectively. Subsequently, using the 137 co-expressed DEGs common to the Con_vs_AD and AD_vs_AZF groups, a PPI network was constructed, revealing the most interactive core genes to be Il1b, Tnf, Tlr4, Tlr2, and Cxcl1 (Figure 5C).

## 3. Discussion

AD is a chronic relapsing skin disease with a complex pathogenesis, involving immune imbalance, dysfunction of the skin barrier, and other factors. Clinically, the main symptoms of AD include erythema, itching, eczematous changes, edema, and lichenification, with severe cases potentially leading to exudation and bacterial infections [26]. The dermatitis score and the frequency of scratching are crucial indicators for assessing the severity of the disease [27]. Consequently, controlling the “itch-scratch” cycle is of paramount importance in the treatment of AD, as it is correlated with clinical symptoms, immune cell infiltration, and a decrease in epidermal thickness [28]. In this study, AD-like reactions were induced in mice by repeatedly applying DNFB to their dorsal skin, and mice in the AD model group exhibited symptoms similar to those mentioned above. Following 15 mg/kg AZF treatment, the erythema, scabbing, and dryness observed on the dorsal skin of AD mice were successfully improved, and a significant reduction was achieved in both the dermatitis score and the number of scratches in AD mice.

Skin, as the primary defense line of the human body, provides an effective barrier function between the organism and the external environment. It not only helps reduce transepidermal water loss but also prevents the invasion of various irritants, antigens, and pathogens. When the skin barrier function is compromised, allergens or pathogens are more likely to penetrate the skin, activating keratinocytes, mast cells, and eosinophils, and prompting them to secrete a series of inflammatory cytokines, thereby triggering an immune response. Hyperkeratosis and inflammatory cell infiltration are another distinct clinical symptom of AD, which includes the infiltration of mast cells, eosinophils, and basophils [29]. In this study, histological analysis using H&E and TB staining revealed thickening of the epidermis and infiltration of mast cells in the model group mice. Compared to the AD model group, treatment with 15–20 mg/kg AZF reduced the epidermal thickness and the degree of mast cell infiltration in mice. Furthermore, atopic dermatitis not only causes skin damage but also affects internal tissues and organs such as the spleen, thymus, bone marrow, and lymph nodes. Among these, the spleen and thymus are two crucial immune organs in the body. The spleen index and thymus index are commonly used to assess the functional status of immune organs. An increase in these two indices is generally associated with the proliferation of immune cells and the activation of immune responses [30,31]. In this study, we observed a significant increase in the spleen and thymus indices of mice after induction with DNFB, indicating that the body is undergoing immune activation or an inflammatory process. After treatment with different doses of AZF, the thymus index of mice was significantly reduced, suggesting that AZF has an inhibitory effect on the excessive immune response induced by AD. However, the effect of AZF on the spleen index of mice was not significant, which may be related to interference from other factors such as genetics and environment.

Adaptive immune cells have been confirmed as major contributors and regulators in AD [32]. During the course of AD, the characteristic infiltration of immune cells in the lesion area undergoes a transition from the acute phase to the chronic phase [33]. During the acute phase of AD, Th2 immune responses predominate. Antigen penetrates through the compromised skin barrier, activating keratinocytes, mast cells, and eosinophils. These cells secrete type 2 cytokines, including IL-4, which are involved in the pathogenesis of AD [34]. The accumulation of IL-4 further activates B cells, prompting them to produce large amounts of IgE. IgE is closely associated with skin itching and impairment of skin barrier function. Allergen-specific IgE, by binding to high-affinity receptors, activates basophils and mast cells, triggering skin inflammation and further damage to the barrier [25,35]. In this study, compared to the control group mice, the AD model group mice exhibited significantly elevated levels of IL-4 and IgE in their serum, accompanied by notable skin barrier damage. Additionally, TSLP plays a crucial role in Th2-type inflammatory responses. Upon binding to dendritic cells, TSLP promotes the differentiation of T cells into Th2 cells, exacerbates the inflammatory response, and induces a Th2-type immune response [36]. With the activation of the inflammatory response, epidermal Langerhans cells secrete large amounts of IL-1β, which, in turn, promotes the secretion of pro-inflammatory mediators such as IL-4 and TNF-α by keratinocytes [37]. As the disease progresses and pro-inflammatory mediators accumulate, the immune response in the lesion area shifts from Th2-dominant to Th1-dominant within 24 to 48 h after the acute phase, secreting abundant Th1-type cytokines such as TNF-α and IFN-γ. Therefore, maintaining the balance between Th1 and Th2 responses is crucial for the development of AD [38]. The elevated serum IFN-γ levels in AD group mice in this study indicated that AD had progressed to a chronic inflammatory state. Concurrently, the serum levels of IL-1β and TSLP remained high in AD group mice. In contrast, compared to the AD group, treatment with 15 and 20 mg/kg AZF reduced the serum levels of IL-4, IFN-γ, IL-1β, TSLP, and IgE in mice. The study results suggest that one of the potential mechanisms by which AZF exerts its anti-AD effects may involve reducing the levels of inflammatory cytokines, thereby restoring the Th1/Th2 immune balance, and modulating the infiltration of inflammatory cells.

Based on the experimental results, although AZF demonstrates anti-AD effects, its therapeutic efficacy lacks dose dependency. This limitation may be the result of multiple factors interacting together. Building upon the research of Stefanelli and colleagues as well as the preliminary work from the laboratory, AZF exhibits not only anti-inflammatory properties but also immunosuppressive effects. Additionally, as the dosage increases, the toxic side effects of AZF also intensify, which may diminish its anti-inflammatory efficacy [22,39]. Pharmacokinetic studies have found that AZF is rapidly distributed to tissues throughout the body after absorption into the bloodstream, leading to insufficient drug concentration at the site of inflammation, thereby affecting the manifestation of its anti-inflammatory effects [40]. Furthermore, early investigations into the biological functions of AZF through oral and intraperitoneal administration revealed limited anti-inflammatory and antibacterial effects [22]. Consequently, in this experiment, the local topical application of AZF was considered more suitable for its development. However, this method of administration is susceptible to various uncontrollable factors such as the behavioral characteristics of mice, and the friction between mice and the bedding as well as the cage and other equipment, which may cause fluctuations in the actual dose of AZF received by the mice during the experiment, thus affecting the uniformity of the experimental results. Despite using CMC-Na as a blank solvent to eliminate the impact of the substrate’s pH on the experiment, differences still exist in the percutaneous absorption efficiency, distribution, and metabolic rate of AZF at different concentrations. These factors can all lead to non-uniformity in the evaluation indicators of the experiment. Nonetheless, the anti-AD effects of AZF are clear.

Following the pharmacodynamic experimental results, subsequent investigations further explored the potential mechanisms of action of AZF in its anti-AD effects. This study employed RNA-Seq to compare the differential gene expression profiles among different treatment groups. The results revealed varying numbers of DEGs among the three groups. GO and KEGG enrichment analyses were conducted on the differential genes, followed by further analysis of gene functions. It was found that the differentially expressed genes were primarily associated with immune and inflammatory responses and enriched in several signaling pathways related to infectious diseases.

Chemokines and cytokines play crucial roles in inflammation and immune responses. Chemokines are a class of small molecular weight proteins that play a key role in immune and inflammatory responses. They regulate the migration and activation of immune cells by binding to specific G protein-coupled receptors, thereby playing a role in inflammatory responses. In the pathogenesis of AD, abnormal changes in the expression of various chemokines and their receptors have been observed. In this study, we identified a group of differentially expressed and upregulated chemokines and their receptors in the Con_vs_AD comparison group, including Ccr1, Ccl3, Cxcl2, Cxcl3, Ccrl2, Cxcr2, Ccl4, Cxcl1, Cxcl5, Ccl8, Ccl6, Ccl9, and Cxcr4. Among them, Cxcl1 and Cxcl3, by binding to their receptor Cxcr2, can promote the migration and activation of immune cells such as neutrophils, monocytes, and T cells, participating in the regulation of inflammatory responses [41,42]. Previous research has indicated that alpha-toxin phenol-soluble modulins (PSMs) secreted by Staphylococcus aureus can significantly induce the expression of pro-inflammatory chemokines such as Cxcl1 and Cxcl3 in human primary keratinocytes, exacerbating skin inflammation [43]. 

Cytokines also play a significant role in the pathogenesis of AD. IL-1β, as a key pro-inflammatory cytokine, triggers inflammatory responses by promoting the proliferation of keratinocytes and activating other immune cells [44]. Research has shown that in AD model mice, the expression level of IL-1β is significantly upregulated, which disrupts the skin barrier and exacerbates skin inflammation by promoting the production of other inflammatory cytokines such as tumor necrosis factor alpha and interleukin 6 [45]. In the Con_vs_AD comparison group of this study, we also observed an upregulation of IL-1β expression, and serological analysis revealed a significant increase in IL-1β levels in AD group mice. Therefore, Cxcl1, Cxcl3, their receptor Cxcr2, as well as IL-1β, may emerge as potential therapeutic targets for AD. Blocking their binding to receptors, utilizing IL-1β inhibitors, or administering IL-1β antibodies may contribute to reducing inflammation and improving AD symptoms. 

Multiple studies have demonstrated that the development of atopic dermatitis (AD) in mice involves multiple signaling pathways, including TLR, NF-κB, JAK-STAT, TNF, MAPK, and others [46,47,48]. Among these, NF-κB serves as a crucial intracellular transcription factor. Once activated during the pathogenesis of AD, it promotes the expression of inflammatory factors such as IL-4 and TNF-α, thereby exacerbating the inflammatory response [49]. Additionally, the TNF signaling pathway, by promoting the activation of keratinocytes and increasing the infiltration of inflammatory cells, interacts with the NF-κB signaling pathway to collectively advance the development of the inflammatory process [47]. The JAK-STAT (Janus kinase-signal transducer and activator of transcription) signaling pathway plays a central role in regulating the activation, proliferation, and differentiation of immune cells. In the pathogenesis of AD, the activation of the JAK-STAT pathway promotes the production of cytokines such as IL-4, IL-5, and TSLP. Furthermore, it is involved in the regulation of the epidermal barrier and the modulation of peripheral nerves associated with itch transmission, thereby contributing to the initiation and progression of AD [13]. 

In this study, we conducted a KEGG enrichment analysis on the DEGs from the Con_vs_AD comparison group and found that the significantly enriched signaling pathways related to signal transduction mainly include cytokine–cytokine receptor interactions, IL-17, NOD-like receptors, chemokines, Toll-like receptors, Staphylococcus aureus infection, NF-κB, TNF, and JAK-STAT signaling pathways. These results emphasize that IL-17, NOD-like receptors, chemokines, Toll-like receptors, Staphylococcus aureus infection, NF-κB, JAK-STAT, and TNF, as well as chemokines and cytokines, are jointly involved in the immune and inflammatory responses induced by DNFB in atopic dermatitis mice. The KEGG enrichment analysis is a powerful tool for identifying the biological pathways that are significantly affected by DEGs, providing insights into the molecular mechanisms underlying the disease.

To uncover the potential molecular mechanisms underlying the therapeutic effects of AZF on AD, this study conducted a detailed analysis of DEGs in the AD_vs_AZF comparison group. In the AD_vs_AZF comparison group, a total of 337 DEGs were identified, including Il1b, Tnf, Tlr4, Tlr2, Cxcl1, etc. These genes were primarily enriched in signaling pathways such as cytokine–cytokine receptor interactions, Toll-like receptors, IL-17, TNF, NF-κB, chemokines, NOD-like receptors, AMPK, B cell receptors, Th17, Staphylococcus aureus infection, and MAPK. Notably, the cytokine–cytokine receptor interaction, NF-κB, TNF, IL-17, NOD-like receptors, chemokines, Toll-like receptors, and Staphylococcus aureus infection pathways were also significantly enriched in the Con_vs_AD comparison group, suggesting that these signaling pathways may play an important role in the treatment of AD with AZF. 

In the Con_vs_AD and AD_vs_AZF comparison groups, a total of 137 DEGs were co-expressed and may be involved in the protective effects of AZF. Among these DEGs, 126 were upregulated in the AD group and downregulated in the AZF group. Notably, Tnf and Il1b were enriched in signaling pathways such as cytokine–cytokine receptor interactions, chemokines, Toll-like receptors, NF-κB, and TNF signaling pathways. These results further support the role of Toll-like receptors, NF-κB, and TNF signaling pathways, as well as cytokines and chemokines, in the treatment of AD with AZF. It is noteworthy that there are interactions between Tnf and Il1b, Il1a, Tlr2, and Tlr4, suggesting that Tnf and Il1b may be key genes in the mechanism of AZF treatment for AD. 

As an inflammatory chemokine, studies have shown that the topical application of linoleic acid–ceramide emollients can significantly reduce the expression levels of Ccl3 in the skin tissue of AD mice [50]. Tlr2 and Tlr4, as primary pattern recognition receptors for peptidoglycan and lipopolysaccharide, respectively, have been implicated in promoting Th2 responses when their signaling is impaired, which can lead to the development of AD. Furthermore, the activation of Tlr2 in keratinocytes not only triggers the production of pro-inflammatory cytokines such as TNF-α and IL-6 but also enhances the tight junction barrier function of the epidermis during pathogen invasion [51]. In immunological processes, Itgam, a member of the G protein-coupled receptor family, forms the CD11b/CD18 complex on the leukocyte surface through binding with the β2 subunit (CD18). Research has demonstrated that cyclosporine A can improve skin rashes in patients with AD by downregulating the expression level of CD11b on the surface of eosinophils [52]. 

Based on the above findings, it is suggested that AZF may alleviate inflammation and immune responses in AD mice through downregulating the expression of Tnf and Il1b genes, thereby modulating the TNF and NF-κB immune signaling pathways. Additionally, other inflammation- and immune-related genes, including Ccl3, Cxcl1, Cxcr2, Cxcl3, Tlr2, Tlr4, and Itgam, also play significant roles in the anti-AD effects of AZF. Similarly, considering the multifaceted physiological functions of AZF, including its anti-inflammatory and immunosuppressive effects, assessing its comprehensive action in the body is undoubtedly a complex and challenging task that cannot be easily concluded. Based on this, the study conducted a preliminary exploration of the potential signaling pathways through which AZF may exert its anti-AD effects.

In summary, the present study comprehensively evaluated the therapeutic effects of AZF on AD using an in vivo animal model through behavioral observations, histopathological analysis, and measurements of inflammatory factor levels. The results indicate that AZF is capable of alleviating AD-related clinical symptoms, including reducing skin lesion scores, scratching frequency, epidermal thickness, and mast cell infiltration in AD mice. Furthermore, AZF restores the inflammatory response and immune imbalance in AD mice by decreasing serum levels of IgE, TSLP, IL-1β, IFN-γ, and IL-4. Meanwhile, tacrolimus ointment, a commonly used clinical treatment for AD, was selected as a positive control, and it was found that 15 mg/kg AZF showed superior efficacy compared to Tac. Through comparative transcriptomic analysis, we further confirmed that the TNF and NF-κB immune signaling pathways may be key mechanisms by which AZF exerts its anti-AD effects. This research is the first to confirm the pharmacological role of AZF in the inflammatory response of AD-like skin lesions and its possible mechanisms of action, providing a new direction for the treatment of AD. However, the therapeutic effect of AZF in human models and its potential for clinical application still require further investigation.

## 4. Materials and Methods 

### 4.1. Drugs and Reagents

Azalomycin F (AZF) was extracted and isolated from the fermentation broth of *Streptomyces hygroscopicus* var. *azalomyceticus* using the methods described in our publication [53]. 2,4-dinitrofluorobenzene (DNFB) was purchased from Shanghai Macklin Biochemical Technology Co., Ltd. (Shanghai, China) Tacrolimus ointment (Tac, 0.1% (g/g)) was obtained from Jiangsu Zhiyuan Pharmaceutical Co., Ltd. (Wuxi, China). Acetone (analytical grade) was purchased from Xilong Chemical Co., Ltd. (Shantou, China). Veet hair removal cream was bought from Reckitt Benckiser Co., Ltd. (London, United Kingdom). Sodium carboxymethyl cellulose (CMC-Na) was sourced from Xilong Chemical Co., Ltd. Dimethyl sulfoxide (DMSO, analytical grade) was purchased from Xilong Chemical Co., Ltd. Tween 80 (analytical grade) was bought from Shanghai Sangon Biotech Co., Ltd. (Shanghai, China), and 4% paraformaldehyde fixative solution was obtained from Beijing Solarbio Science & Technology Co., Ltd. (Beijing, China).

### 4.2. Animals

Female BALB/c mice (6~8 weeks, 18 ± 2 g) were purchased from Changsha Tianqin Biotechnology Co., Ltd. (Changsha, China) [license number: SCXK (Xiang) 2019-0013]. All animals were housed in a 12 h dark/light cycle environment under a controlled temperature of 25 ± 2 °C and under a relative humidity of 40~60%. To avoid the occurrence of stress, mice were acclimatized and fed for one week prior to the start of animal experiments, during which they were allowed to eat and drink freely. All animal experiments were conducted following the guidelines of the Experimental Animal Ethics Committee of Jiangxi Agricultural University, with ethical approval number: JXAULL-2021-08.

### 4.3. Animal Modeling and Grouping

The atopic dermatitis (AD) mouse model was established with reference to the methods in the article, with minor modifications [54,55]. After one week of acclimatization feeding, the mice had their back hair removed using depilating cream, with an area of 2.0 cm × 3.0 cm. Subsequently, the mice were randomly divided into the following 8 groups of 12 mice each: (1) normal control group (Control); (2) AD model group (AD); (3) Tacrolimus group (5 mg/kg Tac); (4) 10 mg/kg Azalomycin F (10 mg/kg AZF); (5) 15 mg/kg Azalomycin F (15 mg/kg AZF); (6) 20 mg/kg Azalomycin F (20 mg/kg AZF); (7) 25 mg/kg Azalomycin F (25 mg/kg AZF); and (8) 30 mg/kg Azalomycin F (30 mg/kg AZF) group. An amount of 0.5 g of CMC-Na powder was accurately weighed and slowly added to 100 mL of ultrapure water preheated to 55–60 °C and then stirred continuously until fully dissolved, to prepare a 0.5% CMC-Na solution, which was then stored in a 4 °C refrigerator for later use. Subsequently, 12, 18, 24, 30, and 36 mg of AZF were precisely weighed and dissolved completely in 240 µL of DMSO, followed by the addition of 120 µL of Tween 80. Then, 5640 µL of 0.5% CMC-Na solution was slowly incorporated, ensuring thorough mixing to create AZF suspensions at concentrations of 10, 15, 20, 25, and 30 mg/kg, ready for subsequent experimental procedures. 

As shown in Figure 1A, mice were sensitized by painting their shaved back skin with 100 μL 0.5% (*v*/*v*) DNFB acetone solution on days 1 and 3. Subsequently, starting from day 8 and every 3 days, the mice were challenged by painting 100 μL 0.3% DNFB acetone solution at the same site to challenge and maintain the AD-like reaction. Control group mice were only treated with an equal volume of acetone solution. After successfully modeling AD mice, the corresponding doses of AZF and Tacrolimus ointment were applied on their backs once daily for a total of 12 days, and the control group and AD group were treated with 0.5% CMC-Na solution.

### 4.4. Evaluation of the Severity of Atopic Dermatitis and Analysis of Organ Index

The skin lesions were observed, and the dermatitis score was evaluated based on the following four symptoms of erythema/bleeding, edema, excoriation/erosion and scales/dryness, which were defined as the sum of individual scores (0, no symptoms; 1, mild; 2, moderate; 3, severe). Additionally, after the final application of DNFB solution stimulation, the mice were placed individually, and the number of times they scratched their back and neck within 15 min was recorded. At the end of the experiment, the spleen and thymus tissues of the mice were weighed to calculate the organ index using the following formula: Organ Index = Organ Weight (mg)/Mouse Weight (g).

### 4.5. Measurement of Inflammatory Factors Release in Serum

At the end of the experiment, the mice were sacrificed, and whole blood was collected from the eyeballs and left at room temperature for 4 h. The upper serum was separated by centrifugation at 3000 rpm for 10 min at 4 °C and stored at −20 °C in the refrigerator for later use. The levels of total IgE, IL-4, IFN-γ, TSLP and IL-1β in serum were detected by a mouse ELISA kit, purchased from Jiangsu Meimian Industrial Co., Ltd. (Yancheng, China), according to the manufacturer’s instruction.

### 4.6. Histopathological Analysis

The dorsal skin tissue was collected and fixed in 4% paraformaldehyde solution for 24 h, followed by dehydration, paraffin embedding, sectioning, staining with hematoxylin and eosin (H&E) and toluidine blue (TB). Finally, the pathological changes, epidermal thickness, and infiltration of inflammatory cells in the skin tissue were observed under an optical microscope.

### 4.7. RNA Extraction, Library Construction, and Sequencing

After the experiment, it was found that the treatment effect of the 15 mg/kg AZF drug group was the best. Therefore, three groups (control group (Con), AD group (AD), and the optimal drug concentration group of 15 mg/kg AZF (AZF)) were selected for further transcriptome sequencing analysis. Nine mice from each group (3 mice/sample, 3 samples/group) were selected for skin tissue samples. The samples were quickly frozen in liquid nitrogen and then stored at −80 °C for subsequent detection. The samples were shipped to Shanghai Personal Biotechnology Co., Ltd. (Shanghai, China) for analysis using dry ice.

According to the manufacturer’s instructions, total RNA was extracted from mouse skin tissue using TRIzol reagent (Solarbio, Beijing, China). Total RNA with a quantity of ≥1 μg was selected, and polyA-tailed mRNA was enriched using Oligo (dT) magnetic beads. Subsequently, the mRNA was randomly fragmented through ion fragmentation. Using the fragmented mRNA as a template and random oligonucleotides as primers, cDNA was synthesized. Subsequently, the double-stranded cDNA was purified, the sticky ends were repaired, and PCR amplification was performed to ultimately construct and obtain the transcriptome library. After normalization, the multiplexed DNA libraries were mixed in equal volumes and gradually diluted and quantified for PE150 sequencing on an Illumina Novaseq sequencer (Shanghai Personal Biotechnology Co., Ltd., Shanghai, China).

### 4.8. Data Analysis of RNA Sequencing

To ensure the accuracy of subsequent bioinformatics analysis, the raw sequencing data were filtered to obtain high-quality sequencing data. The quality-controlled raw data were aligned to the reference genome using the software HiSat2 (v2.1.0). The Read Count values for each gene, representing the original expression levels, were counted using the HTSeq (version 0.9.1) software. For subsequent quantitative analysis, the expression levels were normalized using FPKM (Fragments Per Kilo bases per Million fragments). Differential expression analysis between the two comparison groups was performed using DESeq2 software (v1.38.3), with the screening criteria set as |log2FoldChange| > 1 for the fold change in expression and *p*-value < 0.05 for significance. 

### 4.9. GO Functional Annotation and KEGG Pathway Enrichment Analyses

To gain a deeper understanding of the functions of differentially expressed genes (DEGs) obtained from different treatments, Gene Ontology (GO) annotation and Kyoto Encyclopedia of Genes and Genomes (KEGG) pathway enrichment analysis were performed. The GO functional annotation analysis was conducted using topGO (version 2.50.0), where the *p*-value was calculated using the hypergeometric distribution method (with a significant enrichment standard of *p*-value < 0.05) to identify the significantly enriched GO terms for differential genes (all/up/down), thereby determining the main biological functions that the differential genes perform in the three aspects of BP (Biological Process), MF (Molecular Function), and CC (Cellular Component). The KEGG pathway enrichment analysis was executed utilizing the clusterProfiler software (version 4.6.0), focusing on significantly enriched pathways with *p*-value < 0.05. The online tool Venny 2.1.0 (https://bioinfogp.cnb.csic.es/tools/venny/index.html, accessed 3 October 2024) was used to perform intersection analysis of the two groups of KEGG enrichment pathways to obtain the co-expressed KEGG pathway.

### 4.10. PPI Network Construction and Module Analyses

To establish the protein–protein interaction (PPI) network among DEGs, the STRING database (https://string-db.org/, accessed 20 September 2024) was utilized for protein network interaction analysis. The STRING database, developed by EMBL, is a comprehensive database of protein interactions derived from experiments, data mining, and homology predictions across various species. For two distinct DEGs, the minimum interaction score required was set to 0.90. Subsequently, the PPI network was visualized using the Cytoscape tool (version 3.6.1). To identify core genes and significant networks, the CytoHubba and Molecular Complex Deletion (MCODE) plugins within Cytoscape were downloaded. The core genes were determined using the Maximum Clique Centrality (MCC) ranking method, and the significant network modules were identified with the default parameters of the MCODE plugin.

### 4.11. Statistical Analysis

All experimental data are presented as means ± SD. Graphs were generated using GraphPad Prism 8 software, and statistical analysis was performed using SPSS Statistics 25. One-way analysis of variance (ANOVA) was used for comparisons between multiple groups, with *p* < 0.05 considered significant.

## Figures and Tables

**Table 1:** The summary of 137 co-expressed DEGs in Con_vs_AD and AD_vs_AZF.

Gene_ID	Name	Con_vs_AD	Con_vs_AZF	AD_vs_AZF
ENSMUSG00000036931	Nfkbid	up	-	down
ENSMUSG00000040435	Ppp1r15a	up	-	down
ENSMUSG00000063889	Crem	up	-	down
ENSMUSG00000029373	Pf4	up	-	down
ENSMUSG00000000982	Ccl3	up	up	down
ENSMUSG00000047735	Samd9l	up	-	down
ENSMUSG00000079293	Clec7a	up	up	down
ENSMUSG00000034459	Ifit1	up	up	down
ENSMUSG00000041735	AAdacl4fm3	down	-	up
ENSMUSG00000028874	Fgr	up	-	down
ENSMUSG00000027399	Il1a	up	up	down
ENSMUSG00000020077	Srgn	up	-	down
ENSMUSG00000026480	Ncf2	up	-	down
ENSMUSG00000049130	C5ar1	up	up	down
ENSMUSG00000043953	Ccrl2	up	up	down
ENSMUSG00000029379	Cxcl3	up	up	down
ENSMUSG00000027524	Edn3	down	-	up
ENSMUSG00000046223	Plaur	up	-	down
ENSMUSG00000042265	Trem1	up	up	down
ENSMUSG00000048120	Entpd1	up	up	down
ENSMUSG00000026177	Slc11a1	up	up	down
ENSMUSG00000028859	Csf3r	up	up	down
ENSMUSG00000020120	Plek	up	-	down
ENSMUSG00000050931	Sgms2	up	-	down
ENSMUSG00000027398	Il1b	up	up	down
ENSMUSG00000044103	Il36g	up	up	down
ENSMUSG00000029915	Clec5a	up	-	down
ENSMUSG00000051439	Cd14	up	up	down
ENSMUSG00000054404	Slfn5	up	-	down
ENSMUSG00000057933	Gsta2	down	-	up
ENSMUSG00000037946	Fgd3	up	-	down
ENSMUSG00000048455	Sprr1b	up	up	down
ENSMUSG00000042759	Apobr	up	-	down
ENSMUSG00000033268	Duox1	up	-	down
ENSMUSG00000022534	Mefv	up	-	down
ENSMUSG00000029304	Spp1	up	up	down
ENSMUSG00000037731	Themis2	up	-	down
ENSMUSG00000072620	Slfn2	up	-	down
ENSMUSG00000005413	Hmox1	up	-	down
ENSMUSG00000049685	Cyp2g1	up	up	up
ENSMUSG00000029338	Antxr2	up	-	down
ENSMUSG00000030155	Clec2e	up	up	down
ENSMUSG00000003484	Cyp4f18	up	-	down
ENSMUSG00000035861	Tmprss11b	up	-	down
ENSMUSG00000030144	Clec4d	up	up	down
ENSMUSG00000045502	Hcar2	up	-	down
ENSMUSG00000051920	Rspo2	down	-	up
ENSMUSG00000052270	Fpr2	up	up	down
ENSMUSG00000094733	Csta3	up	up	down
ENSMUSG00000059657	Stfa2l1	up	up	down
ENSMUSG00000019122	Ccl9	up	-	down
ENSMUSG00000051682	Treml4	up	-	down
ENSMUSG00000043939	A530064D06Rik	up	up	down
ENSMUSG00000025804	Ccr1	up	up	down
ENSMUSG00000099398	Ms4a14	up	-	down
ENSMUSG00000050578	Mmp13	up	up	down
ENSMUSG00000079597	Cstdc4	up	up	down
ENSMUSG00000026535	Ifi202b	up	-	down
ENSMUSG00000064345	mt-Nd2	up	-	down
ENSMUSG00000045382	Cxcr4	up	-	down
ENSMUSG00000031504	Rab20	up	-	down
ENSMUSG00000041324	Inhba	up	-	down
ENSMUSG00000064357	mt-Atp6	up	-	down
ENSMUSG00000027202	Slc12a1	up	-	down
ENSMUSG00000039232	Stx11	up	-	down
ENSMUSG00000024529	Lox	up	-	down
ENSMUSG00000022876	Samsn1	up	-	down
ENSMUSG00000064354	mt-Co2	up	-	down
ENSMUSG00000022150	Dab2	up	-	down
ENSMUSG00000024053	Emilin2	up	-	down
ENSMUSG00000112023	Lilrb4b	up	up	down
ENSMUSG00000058755	Osm	up	-	down
ENSMUSG00000032515	Csrnp1	up	-	down
ENSMUSG00000112148	Lilrb4a	up	-	down
ENSMUSG00000065947	mt-Nd4l	up	-	down
ENSMUSG00000026180	Cxcr2	up	-	down
ENSMUSG00000025473	Adam8	up	-	down
ENSMUSG00000029380	Cxcl1	up	-	down
ENSMUSG00000020407	Upp1	up	up	down
ENSMUSG00000064367	mt-Nd5	up	-	down
ENSMUSG00000014599	Csf1	up	-	down
ENSMUSG00000029659	Medag	up	-	down
ENSMUSG00000047562	Mmp10	up	up	down
ENSMUSG00000045362	Tnfrsf26	up	-	down
ENSMUSG00000033777	Tlr13	up	up	down
ENSMUSG00000064368	mt-Nd6	up	-	down
ENSMUSG00000042622	Maff	up	-	down
ENSMUSG00000013974	Mcemp1	up	-	down
ENSMUSG00000030786	Itgam	up	up	down
ENSMUSG00000046203	Sprr2g	up	up	down
ENSMUSG00000018927	Ccl6	up	-	down
ENSMUSG00000032691	Nlrp3	up	up	down
ENSMUSG00000069792	Wfdc17	up	up	down
ENSMUSG00000025383	Il23a	up	-	down
ENSMUSG00000022126	Acod1	up	up	down
ENSMUSG00000020641	Rsad2	up	up	down
ENSMUSG00000014329	Bicc1	up	-	down
ENSMUSG00000027995	Tlr2	up	-	down
ENSMUSG00000070031	Sp140	up	-	down
ENSMUSG00000022026	Olfm4	up	up	down
ENSMUSG00000064363	mt-Nd4	up	-	down
ENSMUSG00000032487	Ptgs2	up	-	down
ENSMUSG00000041754	Trem3	up	up	down
ENSMUSG00000064370	mt-Cytb	up	-	down
ENSMUSG00000067297	Ifit1bl2	up	up	down
ENSMUSG00000035004	Igsf6	up	up	down
ENSMUSG00000059013	Sh2d3c	up	-	down
ENSMUSG00000022514	Il1rap	up	-	down
ENSMUSG00000056054	S100a8	up	up	down
ENSMUSG00000026872	Zeb2	up	-	down
ENSMUSG00000042228	Lyn	up	-	down
ENSMUSG00000052212	Cd177	up	-	down
ENSMUSG00000026271	Gpr35	up	-	down
ENSMUSG00000022651	Retnlg	up	up	down
ENSMUSG00000009633	G0s2	up	-	down
ENSMUSG00000045551	Fpr1	up	up	down
ENSMUSG00000026068	Il18rap	up	-	down
ENSMUSG00000096719	Mrgpra2b	up	up	down
ENSMUSG00000039005	Tlr4	up	-	down
ENSMUSG00000045566	Sprr4	up	-	down
ENSMUSG00000022902	Stfa2	up	up	down
ENSMUSG00000003153	Slc2a3	up	-	down
ENSMUSG00000075122	Cd80	up	-	down
ENSMUSG00000035183	Slc24a5	down	-	up
ENSMUSG00000079652	Garin1a	up	-	down
ENSMUSG00000055775	Myh8	up	up	up
ENSMUSG00000027737	Slc7a11	up	-	down
ENSMUSG00000056529	Ptafr	up	-	down
ENSMUSG00000003555	Cyp17a1	up	up	up
ENSMUSG00000056071	S100a9	up	up	down
ENSMUSG00000005800	Mmp8	up	-	down
ENSMUSG00000064341	mt-Nd1	up	-	down
ENSMUSG00000030142	Clec4e	up	up	down
ENSMUSG00000000204	Slfn4	up	up	down
ENSMUSG00000051498	Tlr6	up	-	down
ENSMUSG00000027360	Hdc	up	up	down
ENSMUSG00000024401	Tnf	up	-	down

**Table 2:** The significantly enriched KEGG pathways of the DEGs in Con_vs_AD and AD_vs_AZF.

Pathway ID	Pathway Name	KEGG Class	Degs Number	Total Number
mmu04060	Cytokine–cytokine receptor interaction	Signaling molecules and interaction	20	289
mmu04061	Viral protein interaction with cytokine and cytokine receptor	Signaling molecules and interaction	12	92
mmu04064	NF-kappa B signaling pathway	Signal transduction	8	105
mmu04668	TNF signaling pathway	Signal transduction	6	113
mmu04657	IL-17 signaling pathway	Immune system	8	93
mmu04621	NOD-like receptor signaling pathway	Immune system	7	205
mmu04062	Chemokine signaling pathway	Immune system	11	190
mmu04625	C-type lectin receptor signaling pathway	Immune system	8	112
mmu04620	Toll-like receptor signaling pathway	Immune system	9	99
mmu04610	Complement and coagulation cascades	Immune system	3	91
mmu04640	Hematopoietic cell lineage	Immune system	7	94
mmu04613	Neutrophil extracellular trap formation	Immune system	8	201
mmu04623	Cytosolic DNA-sensing pathway	Immune system	2	63
mmu04380	Osteoclast differentiation	Development and regeneration	5	124
mmu05332	Graft-versus-host disease	Immune disease	4	56
mmu05323	Rheumatoid arthritis	Immune disease	11	87
mmu05321	Inflammatory bowel disease	Immune disease	7	62
mmu05135	Yersinia infection	Infectious disease: bacterial	5	135
mmu05152	Tuberculosis	Infectious disease: bacterial	10	179
mmu05150	Staphylococcus aureus infection	Infectious disease: bacterial	5	120
mmu05133	Pertussis	Infectious disease: bacterial	8	76
mmu05134	Legionellosis	Infectious disease: bacterial	8	60
mmu05162	Measles	Infectious disease: viral	4	146
mmu05171	Coronavirus disease—COVID-19	Infectious disease: viral	6	235
mmu05164	Influenza A	Infectious disease: viral	6	173
mmu05142	Chagas disease	Infectious disease: parasitic	6	103
mmu05140	Leishmaniasis	Infectious disease: parasitic	8	70
mmu05144	Malaria	Infectious disease: parasitic	4	55
mmu05146	Amoebiasis	Infectious disease: parasitic	8	107
mmu04940	Type I diabetes mellitus	Endocrine and metabolic disease	4	63
mmu04936	Alcoholic liver disease	Endocrine and metabolic disease	7	139
mmu05417	Lipid and atherosclerosis	Cardiovascular disease	12	216
mmu05418	Fluid shear stress and atherosclerosis	Cardiovascular disease	6	145
mmu04217	Necroptosis	Cell growth and death	5	174

## Data Availability

Data are contained within the article.

## Supplementary Materials

The following supporting information can be downloaded at: https://www.mdpi.com/article/10.3390/ijms252312846/s1.

## References

[1] Langan S.M., Irvine A.D., Weidinger S. (2020). Atopic dermatitis. Lancet.

[2] Tian J., Zhang D., Yang Y., Huang Y., Wang L., Yao X., Lu Q. (2023). Global epidemiology of atopic dermatitis: A comprehensive systematic analysis and modelling study. Br. J. Dermatol..

[3] Yang P., Li Y., Li T., Yuan L., Wang S. (2023). Screening differentially expressed genes and the pathogenesis in atopic dermatitis using bioinformatics. Cell Mol. Biol..

[4] Guo Y., Li P., Tang J., Han X., Zou X., Xu G., Xu Z., Wei F., Liu Q., Wang M. (2016). Prevalence of atopic dermatitis in chinese children aged 1–7 ys. Sci. Rep..

[5] Singh M., Pawar M., Bothra A., Choudhary N. (2020). Overzealous hand hygiene during the COVID 19 pandemic causing an increased incidence of hand eczema among general population. J. Am. Acad. Dermatol..

[6] Lan J., Song Z., Miao X., Li H., Li Y., Dong L., Yang J., An X., Zhang Y., Yang L. (2020). Skin damage among health care workers managing coronavirus disease-2019. J. Am. Acad. Dermatol..

[7] Nedoszytko B., Reszka E., Gutowska-Owsiak D., Trzeciak M., Lange M., Jarczak J., Niedoszytko M., Jablonska E., Romantowski J., Strapagiel D. (2020). Genetic and epigenetic aspects of atopic dermatitis. Int. J. Mol. Sci..

[8] Demessant-Flavigny A.L., Connétable S., Kerob D., Moreau M., Aguilar L., Wollenberg A. (2023). Skin microbiome dysbiosis and the role of Staphylococcus aureus in atopic dermatitis in adults and children: A narrative review. J. Eur. Acad. Dermatol. Venereol..

[9] Zhu T.H., Zhu T.R., Tran K.A., Sivamani R.K., Shi V.Y. (2018). Epithelial barrier dysfunctions in atopic dermatitis: A skin-gut-lung model linking microbiome alteration and immune dysregulation. Br. J. Dermatol..

[10] Yang G., Seok J.K., Kang H.C., Cho Y.Y., Lee H.S., Lee J.Y. (2020). Skin barrier abnormalities and immune dysfunction in atopic dermatitis. Int. J. Mol. Sci..

[11] Weidinger S., Beck L.A., Bieber T., Kabashima K., Irvine A.D. (2018). Atopic dermatitis. Nat. Rev. Dis. Primers.

[12] Wu S., Pang Y., He Y., Zhang X., Peng L., Guo J., Zeng J. (2021). A comprehensive review of natural products against atopic dermatitis: Flavonoids, alkaloids, terpenes, glycosides and other compounds. Biomed. Pharmacother..

[13] Huang I.H., Chung W.H., Wu P.C., Chen C.B. (2022). JAK-STAT signaling pathway in the pathogenesis of atopic dermatitis: An updated review. Front. Immunol..

[14] Furue M. (2020). Regulation of filaggrin, loricrin, and involucrin by IL-4, IL-13, IL-17A, IL-22, AHR, and NRF2: Pathogenic implications in atopic dermatitis. Int. J. Mol. Sci..

[15] Fyhrquist N., Muirhead G., Prast-Nielsen S., Jeanmougin M., Olah P., Skoog T., Jules-Clement G., Feld M., Barrientos-Somarribas M., Sinkko H. (2019). Microbe-host interplay in atopic dermatitis and psoriasis. Nat. Commun..

[16] Freitas E., Gooderham M., Torres T. (2022). New topical therapies in development for atopic dermatitis. Drugs.

[17] Li H., Zhang Z., Zhang H., Guo Y., Yao Z. (2021). Update on the pathogenesis and therapy of atopic dermatitis. Clin. Rev. Allergy Immunol..

[18] Yuan G. (2010). Screening of Macrolide-Producing Strains from Mangrove Actinomycetes, and Isolation, Identification and Bioactivity of Metabolites. Ph.D. Thesis.

[19] Yuan G., Hong K., Lin H., She Z., Li J. (2013). New azalomycin F analogs from mangrove *Streptomyces* sp. 211726 with activity against microbes and cancer cells. Mar. Drugs.

[20] Yuan G., Xu L., Xu X., Li P., Zhong Q., Xia H., Hu Y., Li P., Song X., Li J. (2019). Azalomycin F_5a_, a polyhydroxy macrolide binding to the polar head of phospholipid and targeting to lipoteichoic acid to kill methicillin-resistant *Staphylococcus aureus*. Biomed. Pharmacother..

[21] Zhong L., Yang B., Zhang Z., Wang J., Wang X., Guo Y., Huang W., Wang Q., Cai G., Xia F. (2022). Targeting autophagy peptidase ATG4B with a novel natural product inhibitor Azalomycin F_4a_ for advanced gastric cancer. Cell Death Dis..

[22] He S. (2021). Pharmacokinetic, and Anti-Inflammatory, Antibacterial Activity of Azalomycin F. Master’s Thesis.

[23] Yuan G., Wang Y., LIiu X., Xu X., Wang J. (2018). Application of Azalomycin F in the Preparation of Drugs for the Treatment of Atopic Dermatitis.

[24] Yasuda T., Terayama (1963). Clinical effects of anti-candida drug Azalomycin F. Jpn. J. Chemother..

[25] Liu F.T., Goodarzi H., Chen H.Y. (2011). IgE, mast cells, and eosinophils in atopic dermatitis. Clin. Rev. Allergy Immunol..

[26] Min G.Y., Kim E.Y., Hong S., Kim J.H., Kim M., Kim E.J., Park J.H., Sohn Y., Jung H.S. (2021). *Lycopus lucidus* Turcz ameliorates DNCB-induced atopic dermatitis in BALB/c mice. Mol. Med. Rep..

[27] Hong M.R., Lei D., Yousaf M., Chavda R., Gabriel S., Janmohamed S.R., Silverberg J.I. (2021). Reliability and longitudinal course of itch/scratch severity in adults with atopic dermatitis. Dermatitis.

[28] Jang S., Ohn J., Kim J.W., Kang S.M., Jeon D., Heo C.Y., Lee Y.S., Kwon O., Kim K.H. (2020). Caffeoyl-Pro-His amide relieve DNCB-induced atopic dermatitis-like phenotypes in BALB/c mice. Sci. Rep..

[29] Lee Y.S., Ryu H.W., Yang W.K., Park M.H., Park Y.C., Kim D.Y., Kwon H.J., Kim S.Y., Oh S.R., Kim S.H. (2021). A combination of *Olea europaea* leaf extract and *Spirodela polyrhiza* extract alleviates atopic dermatitis by modulating immune balance and skin barrier function in a 1-chloro-2,4-dinitrobenzene-induced murine model. Phytomedicine.

[30] Ma X., Zhang J., Yu T., Yang S., Wen X., Jia S., Wang S., Zhang J. (2024). Mechanism of dihuangyin in treatment of mice with atopic dermatitis by regulating JAK1/STAT3 signaling pathway. Chin. J. Exp. Tradit. Med. Formulae.

[31] Li X., Ye H., Su T., Hu C., Huang Y., Fu X., Zhong Z., Du X., Zheng Y. (2023). Immunity and reproduction protective effects of Chitosan Oligosaccharides in Cyclophosphamide/Busulfan-induced premature ovarian failure model mice. Front. Immunol..

[32] Haddad E.B., Cyr S.L., Arima K., McDonald R.A., Levit N.A., Nestle F.O. (2022). Current and emerging strategies to inhibit type 2 inflammation in atopic dermatitis. Dermatol. Ther..

[33] Scibiorek M., Mthembu N., Mangali S., Ngomti A., Ikwegbue P., Brombacher F., Hadebe S. (2023). IL-4Rα signalling in B cells and T cells play differential roles in acute and chronic atopic dermatitis. Sci. Rep..

[34] Jiang P., Wu Y., Liu L., Zhang L., Song Z. (2022). Combined application of dinitrofluorobenzene and ovalbumin induced AD-like dermatitis with an increase in helper T-cell cytokines and a prolonged Th2 response. BMC Immunol..

[35] Mócsai G., Gáspár K., Dajnoki Z., Tóth B., Gyimesi E., Bíró T., Maródi L., Szegedi A. (2015). Investigation of skin barrier functions and allergic sensitization in patients with Hyper-IgE syndrome. J. Clin. Immunol..

[36] Luo J., Zhu Z., Zhai Y., Zeng J., Li L., Wang D., Deng F., Chang B., Zhou J., Sun L. (2023). The role of TSLP in atopic dermatitis: From pathogenetic molecule to therapeutical target. Mediators Inflamm..

[37] Yamaguchi H.L., Yamaguchi Y., Peeva E. (2023). Role of innate immunity in allergic contact dermatitis: An update. Int. J. Mol. Sci..

[38] Bae S., Jeong N.H., Choi Y.A., Lee B., Jang Y.H., Lee S., Kim S.H. (2023). Lupeol alleviates atopic dermatitis-like skin inflammation in 2,4-dinitrochlorobenzene/*Dermatophagoides farinae* extract-induced mice. BMC Pharmacol. Toxicol..

[39] Stefanelli S., Corti E., Montanini N., Denaro M., Sarubbi E. (1997). Inhibitors of type-I interleukin-1 receptor from microbial metabolites. J. Antibiot..

[40] He S., Zhao W., Li P., Tu W., Hong K., Zhang D., Zhang T., Yuan G. (2021). Pharmacokinetics of azalomycin F, a natural macrolide produced by streptomycete strains, in rats. Molecules.

[41] Cai R., Gong X., Li X., Jiang Y., Deng S., Tang J., Ge H., Wu C., Tang H., Wang G. (2024). Dectin-1 aggravates neutrophil inflammation through caspase-11/4-mediated macrophage pyroptosis in asthma. Respir. Res..

[42] Smith D.F., Galkina E., Ley K., Huo Y. (2005). GRO family chemokines are specialized for monocyte arrest from flow. Am. J. Physiol. Heart Circ. Physiol..

[43] Damour A., Robin B., Deroche L., Broutin L., Bellin N., Verdon J., Lina G., Leclère F.M., Garcia M., Cremniter J. (2021). Phenol-soluble modulins α are major virulence factors of *Staphylococcus aureus* secretome promoting inflammatory response in human epidermis. Virulence.

[44] Lee H.S., Paik J.H., Kwon O.K., Paryanto I., Yuniato P., Ryu H.W., Choi S.H., Oh S.R., Han S.B., Park J.W. (2021). Anti-inflammatory effects of *Lagerstroemia ovalifolia* Teijsm. & Binn. in TNFα/IFNγ-stimulated keratinocytes. Evid. Based Complement. Alternat. Med..

[45] Son Y., Yang W., Park S., Yang J., Kim S., Lyu J.H., Kim H. (2023). The Anti-inflammatory and skin barrier function recovery effects of *schisandra chinensis* in mice with atopic dermatitis. Medicina.

[46] Xiong X., Huang C., Wang F., Dong J., Zhang D., Jiang J., Feng Y., Wu B., Xie T., Cheng L. (2021). Qingxue jiedu formulation ameliorated DNFB-induced atopic dermatitis by inhibiting STAT3/MAPK/NF-κB signaling pathways. J. Ethnopharmacol..

[47] Qu R., Chen X., Hu J., Fu Y., Peng J., Li Y., Chen J., Li P., Liu L., Cao J. (2019). Ghrelin protects against contact dermatitis and psoriasiform skin inflammation by antagonizing TNF-α/NF-κB signaling pathways. Sci. Rep..

[48] Tang L., Li X.L., Deng Z.X., Xiao Y., Cheng Y.H., Li J., Ding H. (2020). Conjugated linoleic acid attenuates 2,4-dinitrofluorobenzene-induced atopic dermatitis in mice through dual inhibition of COX-2/5-LOX and TLR4/NF-κB signaling. J. Nutr. Biochem..

[49] Zhao W., Zhang Y., Li W., Hu Q., Huang H., Xu X., Du B., Li P. (2023). Probiotic-fermented *Portulaca oleracea* L. alleviated DNFB-induced atopic dermatitis by inhibiting the NF-κB signaling pathway. J. Ethnopharmacol..

[50] Zhang J., Xu X., Wang X., Zhang L., Hu M., Le Y., Chen L., Zheng J. (2023). Topical emollient prevents the development of atopic dermatitis and atopic march in mice. Exp. Dermatol..

[51] Lesiak A., Smolewski P., Sobolewska-Sztychny D., Sysa-Jedrzejowska A., Narbutt J. (2012). The role of T-regulatory cells and Toll-like receptors 2 and 4 in atopic dermatitis. Scand. J. Immunol..

[52] Yamada H., Kurashimo S., Chihara J., Matsukura M., Yudate T., Tezuka T. (1999). Overexpression of CD11b on eosinophils in atopic dermatitis: Downregulation by cyclosporin A and upregulation by interleukin 5. Int. Arch. Allergy Immunol..

[53] Yuan G., Lin H., Wang C., Hong K., Liu Y., Li J. (2011). ^1^H and ^13^C assignments of two new macrocyclic lactones isolated from *Streptomyces* sp. 211726 and revised assignments of azalomycins F_3a_, F_4a_ and F_5a_. Magn. Reson. Chem..

[54] Tian T., Chang H., He K., Ni Y., Li C., Hou M., Chen L., Xu Z., Chen B., Ji M. (2019). Fucoidan from seaweed *Fucus vesiculosus* inhibits 2,4-dinitrochlorobenzene-induced atopic dermatitis. Int. Immunopharmacol..

[55] Zheng B.W., Wang B.Y., Xiao W.L., Sun Y.J., Yang C., Zhao B.T. (2022). Different molecular weight hyaluronic acid alleviates inflammation response in DNFB-induced mice atopic dermatitis and LPS-induced RAW 264.7 cells. Life Sci..

