# Supplementary material for: Anti-Atopic Dermatitis Effect of Azalomycin F on 2,4-Dinitrofluorobenzene-Induced Mice and Potential Mechanism
Source: Int J Mol Sci. 2024 Nov 29;25(23):12846. doi: 10.3390/ijms252312846 (PMC11640906; doi:10.3390/ijms252312846)
Supplement: Supplementary file 1 [file ijms-25-12846-s001.zip › ijms-3321755-supplementary.pdf]

Supplementary Materials

Anti-atopic dermatitis effect of azalomycin F on 2,4-dinitrofluorobenzene-induced mice and potential mechanism

Wenjia Zhao[1, 2], Jianping Zhu[1, 3], Xinrong Luo[3], Fengxian Lian[3], Yanli Yang[1], Su He[1], Jinzhou Zhu[3], Ganjun Yuan[1, 3, *]

[1] Biotechnological Engineering Center for Pharmaceutical Research and Development, Jiangxi Agricultural University, Nanchang 330045, China; 18770910458@163.com (W.Z.); zjp13698090160@163.com (J.Z.); yyl01111@163.com (Y.Y.); hs18380172921@163.com (S.H.)

[2] College of Animal Science and Technology, Jiangxi Agricultural University, Nanchang, 330045, China

[3] Laboratory of Natural Medicine and Microbiological Drug, College of Bioscience and Bioengineering, Jiangxi Agricultural University, Nanchang 330045, China; 17336624619@163.com (X.L.); 17848560041@163.com (F.L.); 18226142745@163.com (J.Z.)

[*] Correspondence: gyuan@jxau.edu.cn

This Supplemental Materials includes Fig. S1, Tables S1−4.

Fig. S1. RNA-Seq sequencing analysis.

Table S1. Quality analysis of transcriptome sequencing data.

Table S2. The significant enriched KEGG pathways of the DEGs in Con_vs_AD.

Table S3. The significant enriched KEGG pathways of the DEGs in Con_vs_AZF.

Table S4. The significant enriched KEGG pathways of the DEGs in AD_vs_AZF.

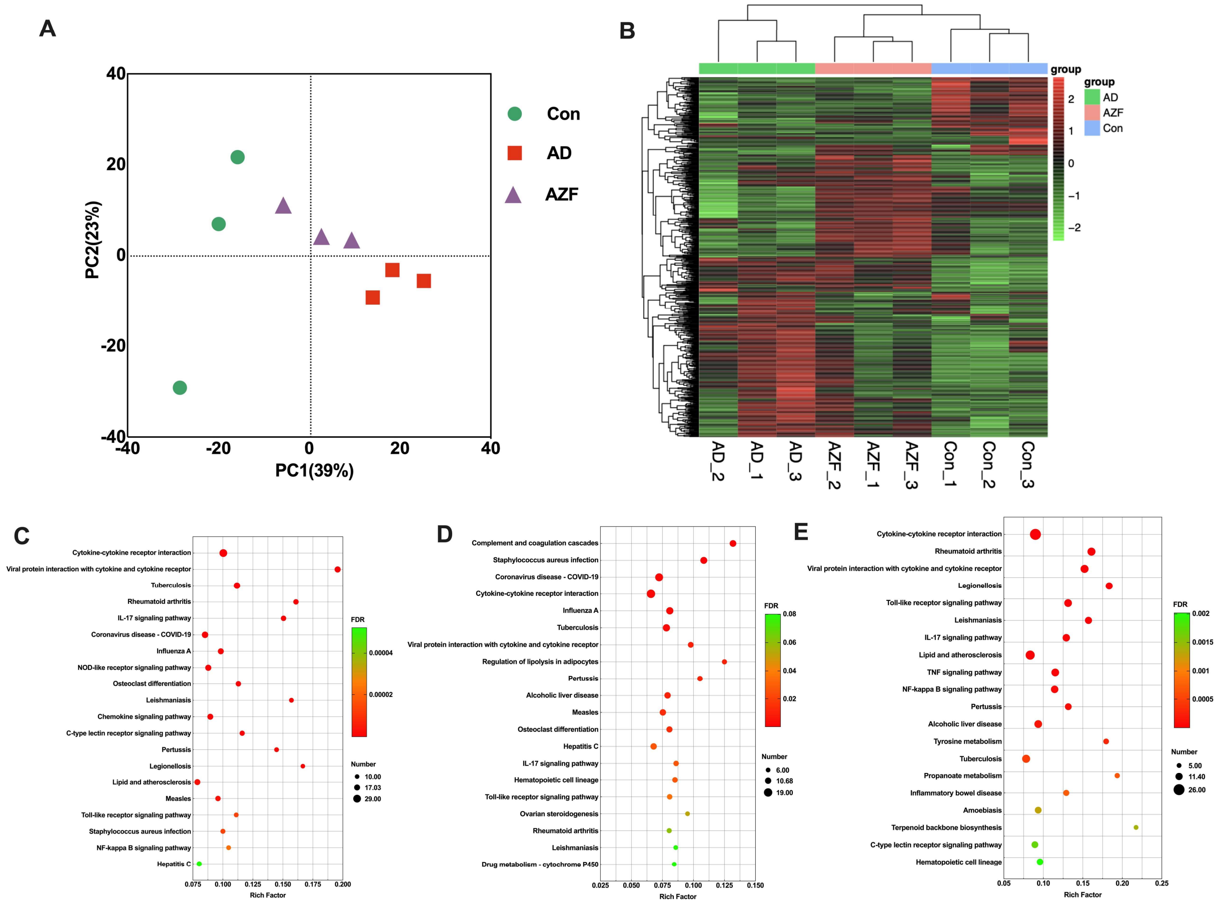

Fig. S1. RNA-Seq sequencing analysis. (A) Principal component analysis of 9 sample relationships. (B) Hierarchical clustering and heat map of DEGs from 9 samples. (C-E) The top 20 pathways with the most significant Kyoto Encyclopedia of Genes and Genomes (KEGG) enrichment in the Con_vs_AD, Con_vs_AZF and AD_vs_AZF group. Con, control group; AD, atopic dermatitis group; AZF, 15 mg/kg azalomycin group.

Table S1. Quality analysis of transcriptome sequencing data

Sample	Raw date No.	Clean date No.	Q20 (%)	Q30 (%)
Con_1	43,003,328	42,191,006	98.38	95.49
Con_2	42,096,038	41,310,492	98.39	95.62
Con_3	47,744,522	46,853,646	98.35	95.43
AD_1	41,647,054	40,883,580	98.42	95.65
AD_2	49,104,000	47,242,836	97.65	93.89
AD_3	38,554,124	37,884,548	98.42	95.66
AZF_1	46,100,978	45,286,108	98.43	95.61
AZF_2	38,578,990	37,904,024	98.41	95.63
AZF_3	36,554.860	35,719,604	98.23	95.20

Note: Q20 means sequence base error < 1%, Q30 means sequence base error < 0.1%.

Table S2. The significant enriched KEGG pathways of the DEGs in Con_vs_AD

Pathway ID	Pathway	KEGG class	*P*-value	DEG number	Total number
mmu04060	Cytokine-cytokine receptor interaction	Signaling molecules and interaction	1.20064E-14	29	289
mmu04061	Viral protein interaction with cytokine and cytokine receptor	Signaling molecules and interaction	1.78642E-14	18	92
mmu05152	Tuberculosis	Infectious disease: bacterial	3.2512E-11	20	179
mmu05323	Rheumatoid arthritis	Immune disease	2.63822E-10	14	87
mmu04657	IL-17 signaling pathway	Immune system	6.6094E-10	14	93
mmu05171	Coronavirus disease - COVID-19	Infectious disease: viral	4.28696E-09	20	235
mmu05164	Influenza A	Infectious disease: viral	7.88333E-09	17	173
mmu04621	NOD-like receptor signaling pathway	Immune system	1.66575E-08	18	205
mmu04380	Osteoclast differentiation	Development and regeneration	3.03782E-08	14	124
mmu05140	Leishmaniasis	Infectious disease: parasitic	3.05633E-08	11	70
mmu04062	Chemokine signaling pathway	Immune system	3.23909E-08	17	190
mmu04625	C-type lectin receptor signaling pathway	Immune system	6.94508E-08	13	112
mmu05133	Pertussis	Infectious disease: bacterial	7.39012E-08	11	76
mmu05134	Legionellosis	Infectious disease: bacterial	7.43078E-08	10	60
mmu05417	Lipid and atherosclerosis	Cardiovascular disease	2.12422E-07	17	216
mmu05162	Measles	Infectious disease: viral	2.41543E-07	14	146
mmu04620	Toll-like receptor signaling pathway	Immune system	1.15025E-06	11	99
mmu05150	Staphylococcus aureus infection	Infectious disease: bacterial	1.16019E-06	12	120
mmu04064	NF-kappa B signaling pathway	Signal transduction	2.07712E-06	11	105
mmu05160	Hepatitis C	Infectious disease: viral	4.98321E-06	13	162
mmu04145	Phagosome	Transport and catabolism	1.02401E-05	13	173
mmu04610	Complement and coagulation cascades	Immune system	2.90147E-05	9	91
mmu04640	Hematopoietic cell lineage	Immune system	3.76526E-05	9	94
mmu05418	Fluid shear stress and atherosclerosis	Cardiovascular disease	4.61982E-05	11	145
mmu05321	Inflammatory bowel disease	Immune disease	9.88359E-05	7	62
mmu05146	Amoebiasis	Infectious disease: parasitic	0.00010455	9	107
mmu04668	TNF signaling pathway	Signal transduction	0.000159194	9	113

mmu04613	Neutrophil extracellular trap formation	Immune system	0.000210842	12	201
mmu05169	Epstein-Barr virus infection	Infectious disease: viral	0.000566794	12	224
mmu04936	Alcoholic liver disease	Endocrine and metabolic disease	0.000742143	9	139
mmu05144	Malaria	Infectious disease: parasitic	0.002695416	5	55
mmu04217	Necroptosis	Cell growth and death	0.003519235	9	174
mmu05167	Kaposi sarcoma-associated herpesvirus infection	Infectious disease: viral	0.004862275	10	217
mmu04623	Cytosolic DNA-sensing pathway	Immune system	0.004868796	5	63
mmu04666	Fc gamma R-mediated phagocytosis	Immune system	0.005805571	6	93
mmu05165	Human papillomavirus infection	Infectious disease: viral	0.008969325	13	352
mmu05142	Chagas disease	Infectious disease: parasitic	0.009444233	6	103
mmu05135	Yersinia infection	Infectious disease: bacterial	0.009611523	7	135
mmu05332	Graft-versus-host disease	Immune disease	0.01662236	4	56
mmu04940	Type I diabetes mellitus	Endocrine and metabolic disease	0.024547229	4	63
mmu05205	Proteoglycans in cancer	Cancer: overview	0.02526717	8	201
mmu04630	JAK-STAT signaling pathway	Signal transduction	0.02773168	7	167
mmu04664	Fc epsilon RI signaling pathway	Immune system	0.028535513	4	66
mmu05163	Human cytomegalovirus infection	Infectious disease: viral	0.03065627	9	248
mmu05202	Transcriptional misregulation in cancer	Cancer: overview	0.033243638	8	212
mmu05221	Acute myeloid leukemia	Cancer: specific types	0.034420348	4	70
mmu05322	Systemic lupus erythematosus	Immune disease	0.040087345	6	143

Table S3. The significant enriched KEGG pathways of the DEGs in Con_vs_AZF

Pathway ID	Pathway	KEGG class	*P*-value	DEG number	Total number
mmu04610	Complement and coagulation cascades	Immune system	1.27784E-06	12	91
mmu05150	Staphylococcus aureus infection	Infectious disease: bacterial	4.39672E-06	13	120
mmu05171	Coronavirus disease - COVID-19	Infectious disease: viral	3.65527E-05	17	235
mmu04060	Cytokine-cytokine receptor interaction	Signaling molecules and interaction	4.74932E-05	19	289
mmu05164	Influenza A	Infectious disease: viral	5.42021E-05	14	173
mmu05152	Tuberculosis	Infectious disease: bacterial	7.86296E-05	14	179
mmu04061	Viral protein interaction with cytokine and cytokine receptor	Signaling molecules and interaction	0.000296112	9	92
mmu04923	Regulation of lipolysis in adipocytes	Endocrine system	0.000313144	7	56
mmu05133	Pertussis	Infectious disease: bacterial	0.000390112	8	76
mmu04936	Alcoholic liver disease	Endocrine and metabolic disease	0.000418457	11	139
mmu05162	Measles	Infectious disease: viral	0.000635268	11	146
mmu04380	Osteoclast differentiation	Development and regeneration	0.0006581	10	124
mmu05160	Hepatitis C	Infectious disease: viral	0.001496171	11	162
mmu04657	IL-17 signaling pathway	Immune system	0.001499664	8	93
mmu04640	Hematopoietic cell lineage	Immune system	0.001606779	8	94
mmu04620	Toll-like receptor signaling pathway	Immune system	0.002236684	8	99
mmu04913	Ovarian steroidogenesis	Endocrine system	0.003501835	6	63
mmu05323	Rheumatoid arthritis	Immune disease	0.004278693	7	87
mmu05140	Leishmaniasis	Infectious disease: parasitic	0.005902579	6	70
mmu00982	Drug metabolism - cytochrome P450	Xenobiotics biodegradation and metabolism	0.006324252	6	71
mmu00830	Retinol metabolism	Metabolism of cofactors and vitamins	0.007731831	7	97
mmu04217	Necroptosis	Cell growth and death	0.007902634	10	174
mmu04064	NF-kappa B signaling pathway	Signal transduction	0.011716622	7	105
mmu05146	Amoebiasis	Infectious disease: parasitic	0.012909713	7	107
mmu05417	Lipid and atherosclerosis	Cardiovascular disease	0.01293717	11	216
mmu05134	Legionellosis	Infectious disease: bacterial	0.013130449	5	60

mmu04062	Chemokine signaling pathway	Immune system	0.014124372	10	190
mmu00900	Terpenoid backbone biosynthesis	Metabolism of terpenoids and polyketides	0.016048593	3	23
mmu04625	C-type lectin receptor signaling pathway	Immune system	0.016271444	7	112
mmu05169	Epstein-Barr virus infection	Infectious disease: viral	0.016578322	11	224
mmu00750	Vitamin B6 metabolism	Metabolism of cofactors and vitamins	0.017771073	2	9
mmu03320	PPAR signaling pathway	Endocrine system	0.018160941	6	89
mmu04270	Vascular smooth muscle contraction	Circulatory system	0.019420683	8	143
mmu00140	Steroid hormone biosynthesis	Lipid metabolism	0.02005047	6	91
mmu04666	Fc gamma R-mediated phagocytosis	Immune system	0.022069627	6	93
mmu04621	NOD-like receptor signaling pathway	Immune system	0.022765652	10	205
mmu04611	Platelet activation	Immune system	0.026784198	7	124
mmu04921	Oxytocin signaling pathway	Endocrine system	0.026839402	8	152
mmu00600	Sphingolipid metabolism	Lipid metabolism	0.027609274	4	49
mmu04750	Inflammatory mediator regulation of TRP channels	Sensory system	0.028901477	7	126
mmu04080	Neuroactive ligand-receptor interaction	Signaling molecules and interaction	0.029060659	15	372
mmu04971	Gastric acid secretion	Digestive system	0.03136408	5	75
mmu04922	Glucagon signaling pathway	Endocrine system	0.035636791	6	104
mmu00500	Starch and sucrose metabolism	Carbohydrate metabolism	0.041696176	3	33
mmu01040	Biosynthesis of unsaturated fatty acids	Lipid metabolism	0.044964043	3	34
mmu04970	Salivary secretion	Digestive system	0.045497311	5	83
mmu04613	Neutrophil extracellular trap formation	Immune system	0.047532886	9	201

Table S4. The significant enriched KEGG pathways of the DEGs in AD_vs_AZF

Pathway ID	Pathway	KEGG class	*P*-value	DEG number	Total number
mmu04060	Cytokine-cytokine receptor interaction	Signaling molecules and interaction	3.6686E-10	26	289
mmu05323	Rheumatoid arthritis	Immune disease	3.35969E-09	14	87
mmu04061	Viral protein interaction with cytokine and cytokine receptor	Signaling molecules and interaction	7.13153E-09	14	92
mmu05134	Legionellosis	Infectious disease: bacterial	4.20432E-08	11	60
mmu04620	Toll-like receptor signaling pathway	Immune system	1.52216E-07	13	99
mmu05140	Leishmaniasis	Infectious disease: parasitic	2.20591E-07	11	70
mmu04657	IL-17 signaling pathway	Immune system	5.64952E-07	12	93
mmu05417	Lipid and atherosclerosis	Cardiovascular disease	7.11659E-07	18	216
mmu04668	TNF signaling pathway	Signal transduction	7.28337E-07	13	113
mmu04064	NF-kappa B signaling pathway	Signal transduction	2.11865E-06	12	105
mmu05133	Pertussis	Infectious disease: bacterial	4.25748E-06	10	76
mmu04936	Alcoholic liver disease	Endocrine and metabolic disease	7.6195E-06	13	139
mmu00350	Tyrosine metabolism	Amino acid metabolism	1.53566E-05	7	39
mmu05152	Tuberculosis	Infectious disease: bacterial	2.65086E-05	14	179
mmu00640	Propanoate metabolism	Carbohydrate metabolism	4.07312E-05	6	31
mmu05321	Inflammatory bowel disease	Immune disease	4.61044E-05	8	62
mmu05146	Amoebiasis	Infectious disease: parasitic	8.86056E-05	10	107
mmu00900	Terpenoid backbone biosynthesis	Metabolism of terpenoids and polyketides	0.000102402	5	23
mmu04625	C-type lectin receptor signaling pathway	Immune system	0.000130193	10	112
mmu04640	Hematopoietic cell lineage	Immune system	0.000166462	9	94
mmu00280	Valine, leucine and isoleucine degradation	Amino acid metabolism	0.000170641	7	56
mmu04062	Chemokine signaling pathway	Immune system	0.000201682	13	190
mmu05418	Fluid shear stress and atherosclerosis	Cardiovascular disease	0.000258082	11	145
mmu04380	Osteoclast differentiation	Development and regeneration	0.000301206	10	124
mmu05142	Chagas disease	Infectious disease: parasitic	0.000333313	9	103

mmu04621	NOD-like receptor signaling pathway	Immune system	0.000423263	13	205
mmu01040	Biosynthesis of unsaturated fatty acids	Lipid metabolism	0.000700376	5	34
mmu05132	Salmonella infection	Infectious disease: bacterial	0.000921619	14	251
mmu00360	Phenylalanine metabolism	Amino acid metabolism	0.00108002	4	22
mmu04932	Non-alcoholic fatty liver disease	Endocrine and metabolic disease	0.001341326	10	150
mmu05208	Chemical carcinogenesis - reactive oxygen species	Cancer: overview	0.001536928	12	207
mmu04146	Peroxisome	Transport and catabolism	0.002314376	7	86
mmu05135	Yersinia infection	Infectious disease: bacterial	0.002320313	9	135
mmu00650	Butanoate metabolism	Carbohydrate metabolism	0.002384726	4	27
mmu00062	Fatty acid elongation	Lipid metabolism	0.003122162	4	29
mmu00600	Sphingolipid metabolism	Lipid metabolism	0.003710825	5	49
mmu05164	Influenza A	Infectious disease: viral	0.003827266	10	173
mmu00410	beta-Alanine metabolism	Metabolism of other amino acids	0.004498309	4	32
mmu00980	Metabolism of xenobiotics by cytochrome P450	Xenobiotics biodegradation and metabolism	0.004500276	6	73
mmu00071	Fatty acid degradation	Lipid metabolism	0.004805049	5	52
mmu05144	Malaria	Infectious disease: parasitic	0.00611096	5	55
mmu05332	Graft-versus-host disease	Immune disease	0.006596758	5	56
mmu04931	Insulin resistance	Endocrine and metabolic disease	0.008539392	7	109
mmu04623	Cytosolic DNA-sensing pathway	Immune system	0.010777186	5	63
mmu00400	Phenylalanine, tyrosine and tryptophan biosynthesis	Amino acid metabolism	0.011617892	2	8
mmu04217	Necroptosis	Cell growth and death	0.011984204	9	174
mmu05162	Measles	Infectious disease: viral	0.012672889	8	146
mmu04975	Fat digestion and absorption	Digestive system	0.012881956	4	43
mmu04672	Intestinal immune network for IgA production	Immune system	0.012881956	4	43
mmu00190	Oxidative phosphorylation	Energy metabolism	0.014044832	7	120
mmu00620	Pyruvate metabolism	Carbohydrate metabolism	0.015051545	4	45
mmu04152	AMPK signaling pathway	Signal transduction	0.015907645	7	123
mmu00340	Histidine metabolism	Amino acid metabolism	0.017256502	3	26

mmu00982	Drug metabolism - cytochrome P450	Xenobiotics biodegradation and metabolism	0.017435067	5	71
mmu05020	Prion disease	Neurodegenerative disease	0.019295104	11	253
mmu05415	Diabetic cardiomyopathy	Cardiovascular disease	0.020830973	9	191
mmu01523	Antifolate resistance	Drug resistance: antineoplastic	0.023153125	3	29
mmu04662	B cell receptor signaling pathway	Immune system	0.023912514	5	77
mmu04659	Th17 cell differentiation	Immune system	0.024433233	6	105
mmu04613	Neutrophil extracellular trap formation	Immune system	0.027840297	9	201
mmu05171	Coronavirus disease - COVID-19	Infectious disease: viral	0.028587131	10	235
mmu00630	Glyoxylate and dicarboxylate metabolism	Carbohydrate metabolism	0.030009013	3	32
mmu00020	Citrate cycle (TCA cycle)	Carbohydrate metabolism	0.03250642	3	33
mmu05204	Chemical carcinogenesis - DNA adducts	Cancer: overview	0.033219703	5	84
mmu04066	HIF-1 signaling pathway	Signal transduction	0.033432917	6	113
mmu03320	PPAR signaling pathway	Endocrine system	0.041079274	5	89
mmu05150	Staphylococcus aureus infection	Infectious disease: bacterial	0.042876907	6	120
mmu05143	African trypanosomiasis	Infectious disease: parasitic	0.043540267	3	37
mmu00140	Steroid hormone biosynthesis	Lipid metabolism	0.044512945	5	91
mmu04610	Complement and coagulation cascades	Immune system	0.044512945	5	91
mmu04913	Ovarian steroidogenesis	Endocrine system	0.044916367	4	63
mmu04940	Type I diabetes mellitus	Endocrine and metabolic disease	0.044916367	4	63
mmu00983	Drug metabolism - other enzymes	Xenobiotics biodegradation and metabolism	0.046292458	5	92
mmu05010	Alzheimer disease	Neurodegenerative disease	0.048749848	13	366
mmu04010	MAPK signaling pathway	Signal transduction	0.049447115	11	294
